# Supplementary material for: Data from the National Educational Panel Study (NEPS) in Germany: Educational Pathways of Students in Grade 5 and Higher
Source: J Open Psychol Data. 2023 Feb 7;11:3. doi: 10.5334/jopd.79 (PMC12270087; doi:10.5334/jopd.79)
Supplement: Appendix Table A1. — Overview of NEPS SC3 survey program. [file jopd-11-79-s1.pdf]

Appendix

Table A1.
Overview of NEPS SC3 survey program.

Competences	Wave 1 (2010/11)	Wave 2 (2011/12)	Wave 3 (2012/13)	Wave 4 (2013/14)	Wave 5 (2014/15)	Wave 6 (2015)	Wave 7 (2016)	Wave 8 (2016/17)	Wave 9 (2017/18)
**Domain-General Competences**									
- Reasoning	S					S			
- Perceptual speed	S					S			
**Domain-Specific Competences**									
- Reading competence*	S		S			S			S
- Reading speed	S				S				
- Listening comprehension at word level: receptive vocabulary		S							
- Listening comprehension at text or discourse level						S			
- Mathematical competence*	S		S		S				S
- Scientific competence*		S			S			S	
- Language competence Languages of origin Russian and Turkish (L1)			S			S			

**Stage-Specific Competences**									
- Orthography	S		S		S				
- Language competence Foreign language English (L2)							S		S
- Scientific thinking									S
**Meta-Competences**									
- Declarative Metacognition		S				S			
- ICT Literacy*		S			S				S

Note. S = Student
** Competence scores are linked over time.*
Last competence measurement was in Wave 9.

Learning Environments	Wave 1 (2010 /11)	Wave 2 (2011 /12)	Wave 3 (2012 /13)	Wave 4 (2013 /14)	Wave 5 (2014 /15)	Wave 6 (2015)	Wave 7 (2016)	Wave 8 (2016 /17)	Wave 9 (2017 /18)	Wave 10 (2018 /19)	Wave 11 (2019 /20)	Wave 12 (2020 /21)
**Learning opportunities in formal learning environments**												
- Instructional quality	S, P, T	P, T	S, T	S, T	S, T							
- Characteristics of schools	T, H		T, H	S, H	T, H	P	S, H	S, H	S	S	S	S
- Characteristics of classes	T	T	T	T	T		S, T					
- Lessons in higher education institution									S	S	S	S
- Characteristics of higher education institution										S	S	S
- Quality of study courses										S	S	S
- Quality of vocational training			S		S			S	S			
- Students´ budget of time for learning										S	S	S
- Characteristics, attitudes, educational design of educational staff	T	T, H	T, H	T, H	T, H		T, H	H				
- Instructional design & lessons		P, T	P, T	T	T			S	S	S	S	S
**Learning opportunities in nonformal/informal learning environments**												
- Quality of courses	S, P			S	S		S	S	S	S	S	S
- Sports	S			S			S			S		S
- Quality of internship						S						
- Private tutoring		P	P			P	S	S				

- Other informal learning opportunities						S						
- ICT/Digital media usage											S	
**Family/Home Learning Environment**												
- Homework	S		S									
- School based parental involvement	P	P	P	S, P		P						
**Transitions**												
- Support in transition into vocational training/ vocational preparation	T, H				T		T	S	S	S	S	S
- Perceived barriers and problems in transition	S									S	S	S
- Measures of delivering learning environment										S	S	S
- Measures of including learning environment											S	

Note. S = Student, P = Parent, T = Teacher, H = Head of school.
Information is taken from the tool Variable Search (https://www.neps-data.de/Data-Center/Overview-and-Assistance/NEPSplorer).

**Motivation & personality**	**Wave 1 (2010 /11)**	**Wave 2 (2011 /12)**	**Wave 3 (2012 /13)**	**Wave 4 (2013 /14)**	**Wave 5 (2014 /15)**	**Wave 6 (2015)**	**Wave 7 (2016)**	**Wave 8 (2016 /17)**	**Wave 9 (2017 /18)**	**Wave 10 (2018 /19)**	**Wave 11 (2019 /20)**	**Wave 12 (2020 /21)**
**Interest**												
- General orientations of interest		S	S	P		S			S	S	S	S
- Topic-related interest		S				S						
- Subject-related interest	S	S										
**Professional orientation**												
- Desired working conditions				S	S							
**Self-concept**												
- Self-concept verbal	S				S						S	
- Self-concept math	S		S		S	S					S	
- Self-concept German			S			S						
- Self-concept school	S				S				S	S	S	S
- Academic self-concept										S	S	S
- Self-efficacy expectations			S									
- Self-esteem	S				S				S	S	S	S
- Reading self-concept	S		S	S		S						
- Orthography self-concept	S											

**Motivation**												
- Object-related intrinsic motivation German				S				S				
- Object-related intrinsic motivation Mathematics				S				S	S	S	S	S
- Competition-related				S				S				
- extrinsic motivation												
- Profession-related				S				S	S	S	S	S
- extrinsic motivation												
- Reading motivation	S	S	S	S	S	S						
- Orthography motivation	S											
- Optimization Strategies							S	S				
- Object-related intrinsic motivation								S				
**Effort**												
- Effort math								S				
- Effort German								S	S	S	S	S
- Effort school								S				
- Flexible goal adjustment & Tenacious goal pursuit (TenFlex)							S	S				

**Personality**												
- Gender roles	P	S				P	S					
- Big Five		P	S, P	P	S	P				S		
- Willingness to take risks					S		S	S	S			S
- Self-evaluated patience									S	S		S
**Coping with everyday school life**												
- Autonomy		P	P	P								
- Joy of learning		P	P	P		P						
- Willingness to make an effort		P	P	P		P						
- Social integration into class		P	P	P								
- Accuracy		P	P	P		P						
**Assessment of competencies**												
- Parents about target child		P	P	P								
**Social competence**												
- Behavioural strengths and difficulties		S, P		P		S, P						
- Interpersonal competencies									S	S	S	S

Note. S = Student, P = Parent, T = Teacher, H = Head of school.
Information is taken from the tool Variable Search (https://www.neps-data.de/Data-Center/Overview-and-Assistance/NEPSplorer).

**Educational decisions**	**Wave 1 (2010/11)**	**Wave 2 (2011/12)**	**Wave 3 (2012/13)**	**Wave 4 (2013/14)**	**Wave 5 (2014/15)**	**Wave 6 (2015)**	**Wave 7 (2016)**	**Wave 8 (2016/17)**	**Wave 9 (2017/18)**	**Wave 10 (2018/19)**	**Wave 11 (2019/20)**	**Wave 12 (2020/21)**
**Rational Choice**												
- Subjective probability of success of the educational option		S, P		S, P	S	S	S	S	S	S	S	S
- Benefits of the educational option		S, P		S, P		S	S	S	S	S	S	S
- Costs of the educational option		S, P		S, P		S	S	S	S	S	S	S
- Status maintenance		S, P		S, P		S	S	S	S	S	S	S
**Bounded rationality/norms/framing**												
- Aspirations	S	S, P	S, P	S, P	S	S, P	S	S	S	S	S	S
- Information		S	P	S, P	S	S, P	S	S	S	S	S	S
- Generalized attitude towards education			S, P					S	S	S	S	S
- Intended subsequent occupation					S	S	S	S	S	S	S	S
- Future career plans							S	S	S	S	S	S
- Future planning		S, P		S, P	S	S	S	S	S	S	S	S
**Cultural capital**												
- Objectified cultural capital	S, P	S	S, P	S	S	P						
- Incorporated cultural capital	S, P				S	P						

- Reading (quantity & quality)	S, P	S	S	S	S	P						
**Social capital**												
- Normative influence (reference groups)		S	P	S	S	S	S	S	S	S	S	S
- Global measurement of access to resources		P		P		S	S	S	S	S	S	S
- Position generator						P		S			S	
- Social capital within the family	P		S, P			P		S	S	S	S	S
- Social closure and social capital between family and educational institutions			P									
**Academic and social integration of university students**												
- Academic integration of students										S	S	S

Note. S = Student, P = Parent, T = Teacher, H = Head of school.
Information is taken from the tool Variable Search (https://www.neps-data.de/Data-Center/Overview-and-Assistance/NEPSplorer).

**Migration**	**Wave 1 (2010 /11)**	**Wave 2 (2011 /12)**	**Wave 3 (2012 /13)**	**Wave 4 (2013 /14)**	**Wave 5 (2014 /15)**	**Wave 6 (2015)**	**Wave 7 (2016)**	**Wave 8 (2016 /17)**	**Wave 9 (2017 /18)**	**Wave 10 (2018 /19)**	**Wave 11 (2019 /20)**	**Wave 12 (2020 /21)**
**Migration biography**												
- Migration background (S, P)	S, P	S, P	S, P	S, P	S, P	P		S	S			S
- Migration biography (S, P)	S	S, P	S, P	S, P	S	P			S	S	S	S
- Citizenship (S, P)	P	S, P	S, P	S, P	S, P	P			S		S	
- Migration intentions		S		P		P	S	S	S	S		
**Language**												
- Language(s) of origin	S, P	S, P	S, P	S	S	P			S			S
- Learning German	S	P	P	P								
- Subjective competence Language of origin	S		S, P		S			S		S		S
- Subjective competence German	S		S, P		S			S		S		S
- Language use	S, P	P	S		S			S		S		S
- Language use media	S		S, P		S			S		S		S
- Language training		P	S, P		S		S					
**Cultural orientation**												
- Identity - Acculturation orientation - Country of origin		S	S	S, P	S		S	S	S	S		S
- Identity - Acculturation orientation - Germany			S	S, P		S	S	S	S	S	S	S

- Cultural habits				S, P					S	S		
**Religion and religiosity**												
- Religiousness		S, P									S	
- Religious affiliation		S, P									S	
- Religious practice		S, P									S	
**Perceived discrimination**												
- Perceived group-specific discrimination							S	S				S
- Perceived personal discrimination								S				S
**Migration-specific learning environment**												
- Migration background educational staff	T, H	T, H	T, H	T, H	T, H		T, H	H	H			
- Migration background pees within school/class	T, H	S, T	T, H	S	S, T, H	S	S, T	S, H	S, H	S	S	
- Language training (offer)	H	H	H	H	H		H					
- Language(s) of origin - educational staff	T	T	T	T	T		T					
- Language use educators	T	T	T	T	T		T					
- Other support measures	H		H		H							
- Multicultural beliefs			T				T					

Note. S = Student, P = Parent, T = Teacher, H = Head of school.
Information is taken from the tool Variable Search (https://www.neps-data.de/Data-Center/Overview-and-Assistance/NEPSplorer).

**Returns to education**	**Wave 1 (2010 /11)**	**Wave 2 (2011 /12)**	**Wave 3 (2012 /13)**	**Wave 4 (2013 /14)**	**Wave 5 (2014 /15)**	**Wave 6 (2015)**	**Wave 7 (2016)**	**Wave 8 (2016 /17)**	**Wave 9 (2017 /18)**	**Wave 10 (2018 /19)**	**Wave 11 (2019 /20)**	**Wave 12 (2020 /21)**
**Monetary returns**												
- Earned income								S	S	S	S	S
- Other subsidies								S	S	S	S	S
**Satisfaction**												
- Life satisfaction	S	S	S	S	S	S	S	S	S	S	S	S
- Satisfaction with life domains	S	S	S	S	S	S	S	S	S	S	S	S
**Political participation**												
- Political orientation				S				S	S	S	S	S
- Voting behaviour										S		
- Actual participation in political actions								S	S	S	S	S
- Potential participation in political actions								S				
- Understanding of Democracy											S	
**Social participation**												
- Trust: institutional, social, group-specific								S			S	S
- Social belonging								S	S	S	S	S
- Memberships						S		S				

**Deviant behaviour**												
- Peer Norms						S						
**Health**												
- Health status	S	S, P	S, P	S, P	S	S, P	S	S	S	S	S	S
- Health behaviour	S		S	S, P	S	S	S	S	S	S		S
- Health in early childhood		P	P	P								
**Expectations in terms of returns**												
- Expected income					S		S	S	S	S	S	S
**Job quality**												
- Characteristics of job										S	S	S
- Work-Life-Conflict				P		P						

Note. S = Student, P = Parent, T = Teacher, H = Head of school.
Information is taken from the tool Variable Search (https://www.neps-data.de/Data-Center/Overview-and-Assistance/NEPSplorer).
